# Supplementary material for: Testing the cultural-invariance hypothesis: A global analysis of the relationship between scientific knowledge and attitudes to science
Source: PLoS One. 2024 Feb 5;19(2):e0296860. doi: 10.1371/journal.pone.0296860 (PMC10843117; doi:10.1371/journal.pone.0296860)
Supplement: S1 File — (DOCX) [file pone.0296860.s001.docx]

Supplementary information

TABLE S1 Item Response Theory 2-parameter logit model – general attitude to science

|  | Difficulty | SE | Discrimination | SE |
| --- | --- | --- | --- | --- |
| In general, do you think the work that scientists do benefits most, some, or very few people in this country? A lot (1), Some (0), Not much (0), Not at all (0). (attitude 1) | 0.406 | 0.006 | 1.706 | 0.025 |
| In general, do you think the work that scientists do benefits people like you in this country? Yes (1), No (0) (attitude 2) | -0.596 | 0.004 | 5.102 | 0.301 |
| Overall, do you think that science and technology will help improve life for the next generation? Yes (1), No (0) (attitude 3) | -1.668 | 0.013 | 1.621 | 0.021 |

FIG S1 Item characteristic curves from IRT mode- general attitude to science


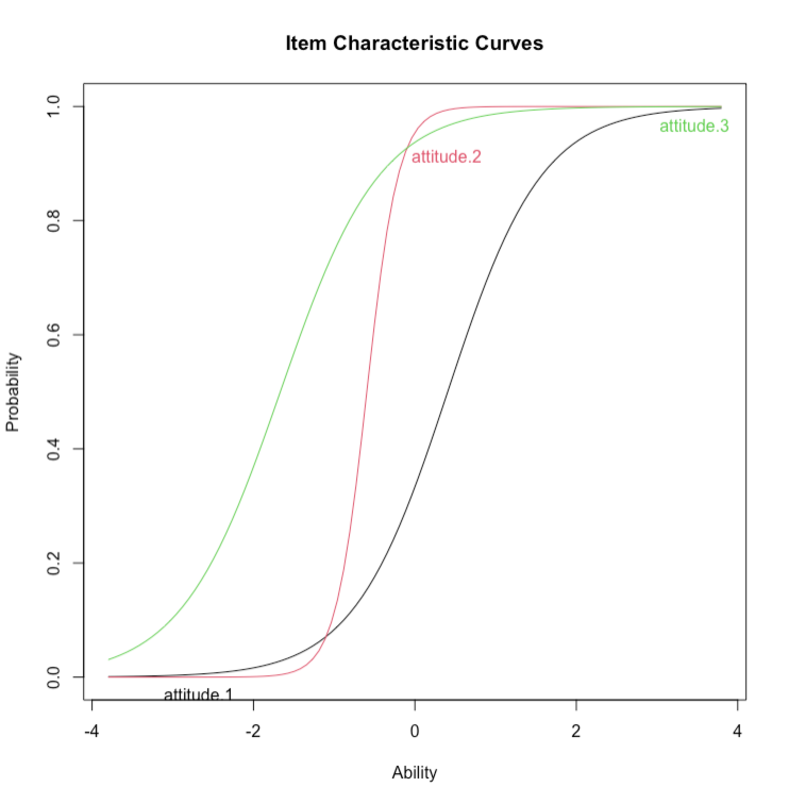


TABLE S2 Item Response Theory 2-parameter logit model – science literacy

|  | Difficulty | SE | Discrimination | SE |
| --- | --- | --- | --- | --- |
| Do you think studying diseases is a part of science? Yes (1), No (0) (true. 1) | -1.661 | 0.022 | 1.511 | 0.035 |
| How much did you understand the meaning of ‘science’ and ‘scientists’ that was just read? A lot (1), Some (0), not much (0), not at all (0) (true.3) | 0.836 | 0.012 | 1.676 | 0.045 |
| Before today, had you ever heard of a vaccine? Yes (1), No (0) (true.4) | -2.392 | 0.034 | 1.030 | 0.019 |

FIG S2 Item characteristic curves from IRT mode- science literacy


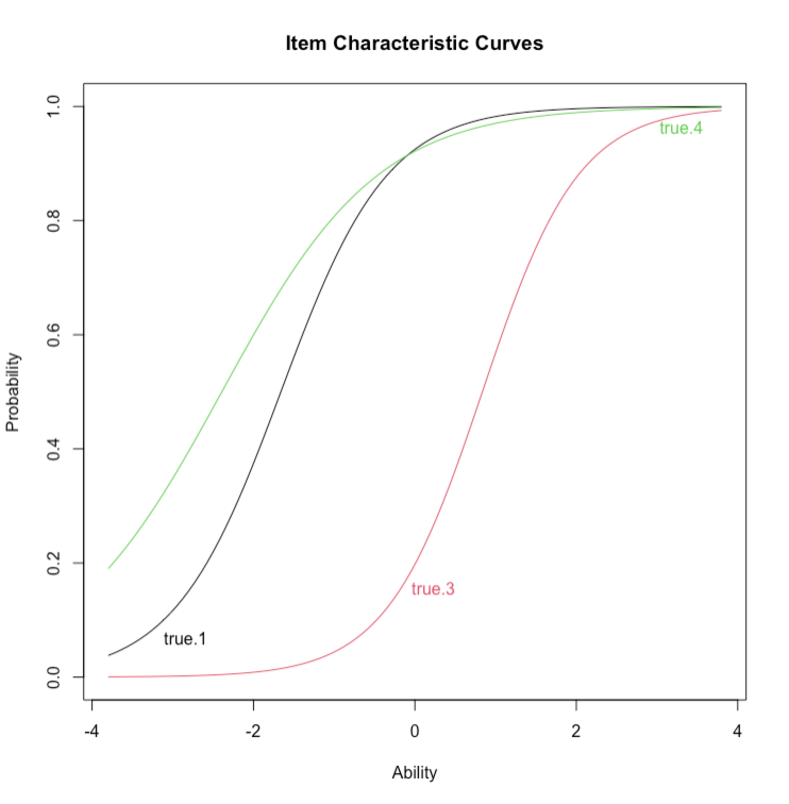


TABLE S3 Item Response Theory 2-parameter logit model – science literacy (including poetry item

|  | Difficulty | SE | Discrimination | SE |
| --- | --- | --- | --- | --- |
| Do you think studying diseases is a part of science? Yes (1), No (0) | -1.735 | 0.020 | 1.398 | 0.027 |
| How much did you understand the meaning of ‘science’ and ‘scientists’ that was just read? A lot (1), Some (0), not much (0), not at all (0) | 0.785 | 0.010 | 1.924 | 0.050 |
| Before today, had you ever heard of a vaccine? Yes (1), No (0) | -2.448 | 0.034 | 0.997 | 0.018 |
| Do you think poetry is a part of science? (Yes(1), No(0) | -0.696 | 0.017 | 0.479 | 0.009 |

FIG S3 Item characteristic curves from IRT mode- science literacy (4 items)

**
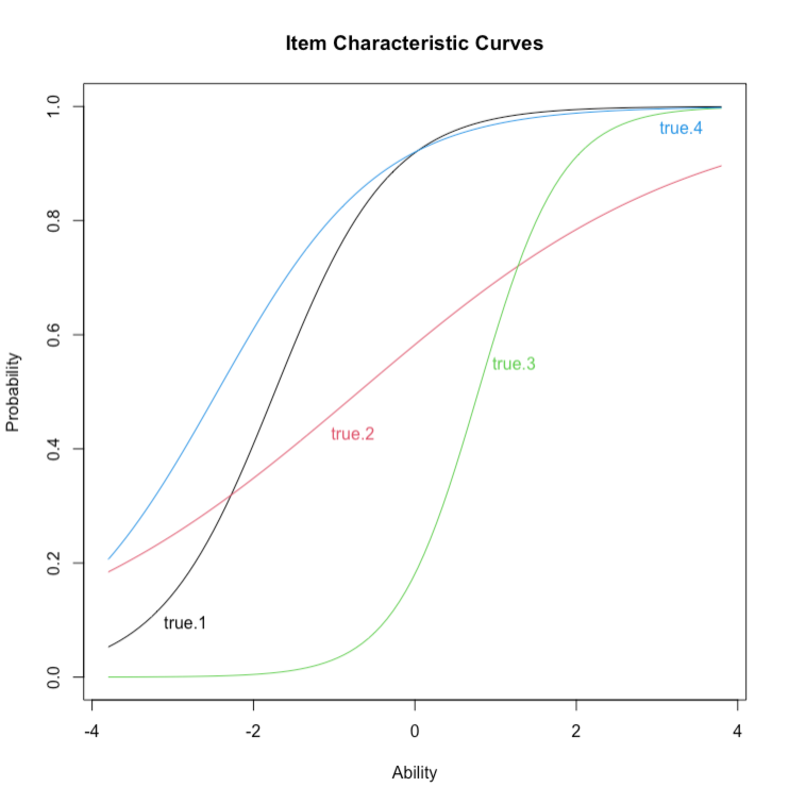
**

**Fig S4** **Distribution of general attitudes to science across countries**

**
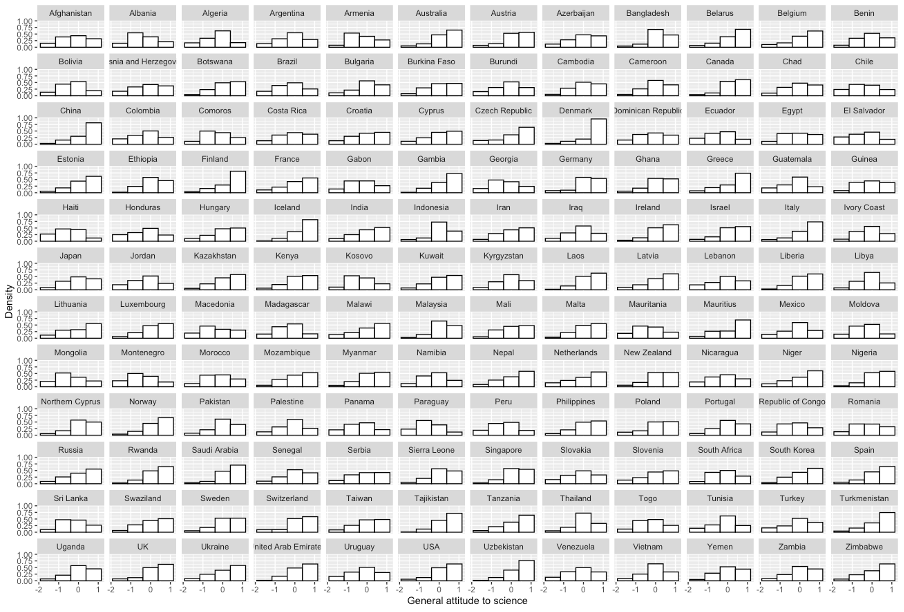
**

**Fig S5** **Distribution of science literacy across countries**

**
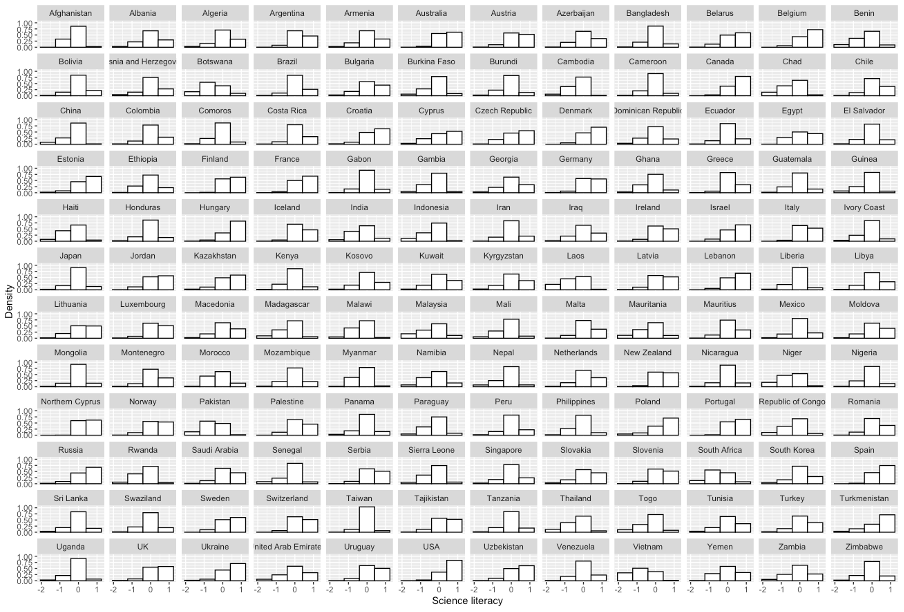
**

**Fig S6** **Distribution of self-assessed knowledge across countries**

**
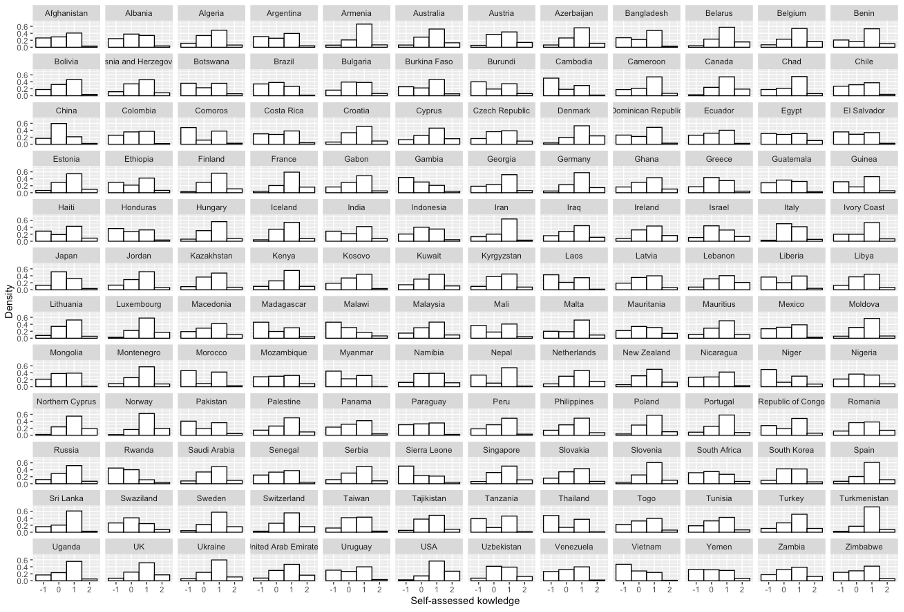
**

TABLE S4 Country mean scores for science literacy and self-assessed knowledge (ranked by literacy)

| **Country** | **Science literacy (%)** | **Self-assessed knowledge (%)** |
| --- | --- | --- |
| Hungary | 69.2 | 8.1 |
| USA | 67.1 | 22.0 |
| Canada | 63.5 | 16.6 |
| Belgium | 59.2 | 16.4 |
| Estonia | 57.9 | 11.8 |
| Turkmenistan | 57.3 | 6.9 |
| Poland | 57.0 | 9.1 |
| Australia | 56.3 | 13.6 |
| Denmark | 55.7 | 21.3 |
| Finland | 55.4 | 11.0 |
| Ukraine | 55.1 | 8.4 |
| Russia | 54.6 | 6.6 |
| Lebanon | 54.5 | 17.2 |
| Spain | 53.8 | 9.2 |
| Israel | 53.1 | 13.1 |
| France | 51.0 | 15.0 |
| New Zealand | 49.9 | 11.5 |
| Uzbekistan | 49.9 | 12.2 |
| Kazakhstan | 49.7 | 6.7 |
| Sweden | 46.8 | 12.0 |
| Belarus | 46.2 | 11.2 |
| UK | 46.0 | 14.0 |
| Norway | 45.4 | 17.0 |
| Croatia | 45.3 | 6.3 |
| Germany | 44.9 | 15.0 |
| Jordan | 44.9 | 5.5 |
| Czech Republic | 44.9 | 9.2 |
| Northern Cyprus | 44.7 | 15.0 |
| Latvia | 44.4 | 5.9 |
| Uruguay | 43.8 | 3.5 |
| Portugal | 43.2 | 4.9 |
| Ireland | 41.6 | 15.2 |
| Slovenia | 40.9 | 8.4 |
| Austria | 40.5 | 12.0 |
| Serbia | 40.1 | 6.5 |
| Italy | 39.8 | 5.4 |
| Slovakia | 39.6 | 8.8 |
| Tajikistan | 39.4 | 6.5 |
| Lithuania | 38.1 | 5.4 |
| Luxembourg | 37.5 | 13.3 |
| Iceland | 37.2 | 6.5 |
| Egypt | 36.8 | 10.1 |
| Cyprus | 36.8 | 11.0 |
| Switzerland | 35.6 | 11.5 |
| Netherlands | 35.4 | 13.9 |
| Bulgaria | 35.1 | 5.6 |
| Palestine | 34.6 | 7.9 |
| Chile | 34.2 | 3.9 |
| Argentina | 33.7 | 3.7 |
| Moldova | 33.2 | 6.7 |
| Saudi Arabia | 32.5 | 8.5 |
| Romania | 31.4 | 10.7 |
| Kuwait | 30.7 | 10.2 |
| Macedonia | 30.0 | 7.7 |
| Costa Rica | 28.9 | 5.2 |
| Malta | 28.6 | 8.0 |
| Armenia | 27.7 | 5.9 |
| Kyrgyzstan | 27.4 | 5.9 |
| Turkey | 27.0 | 9.0 |
| South Korea | 26.8 | 5.6 |
| Georgia | 26.2 | 4.3 |
| United Arab Emirates | 25.7 | 14.9 |
| Montenegro | 25.6 | 6.5 |
| Greece | 25.4 | 4.6 |
| Tunisia | 24.6 | 4.8 |
| Albania | 24.1 | 3.6 |
| Iraq | 23.7 | 8.8 |
| Azerbaijan | 22.8 | 8.8 |
| Kosovo | 22.6 | 2.8 |
| Mauritius | 22.4 | 7.3 |
| Yemen | 22.1 | 5.1 |
| Colombia | 22.0 | 1.7 |
| Algeria | 21.7 | 4.6 |
| Brazil | 21.7 | 1.8 |
| Bosnia and Herzegovina | 20.9 | 6.6 |
| Libya | 19.7 | 5.1 |
| Zambia | 19.2 | 9.3 |
| Mexico | 18.7 | 3.3 |
| Venezuela | 18.5 | 2.0 |
| Bolivia | 18.5 | 4.8 |
| Singapore | 18.4 | 10.4 |
| Dominican Republic | 17.4 | 3.5 |
| Iran | 17.3 | 3.2 |
| Ecuador | 16.4 | 2.8 |
| Peru | 16.2 | 2.9 |
| El Salvador | 15.0 | 2.7 |
| Zimbabwe | 14.7 | 5.3 |
| Mozambique | 14.7 | 10.1 |
| Swaziland | 14.2 | 6.2 |
| Ethiopia | 13.8 | 5.1 |
| Mongolia | 13.5 | 1.7 |
| Morocco | 13.3 | 3.2 |
| Honduras | 12.9 | 3.6 |
| Tanzania | 12.8 | 1.3 |
| Sri Lanka | 12.6 | 2.4 |
| Panama | 12.4 | 4.0 |
| Nicaragua | 12.1 | 2.3 |
| Bangladesh | 11.9 | 2.5 |
| Namibia | 11.2 | 10.8 |
| Guatemala | 11.0 | 2.2 |
| Burundi | 11.0 | 6.2 |
| Malaysia | 10.9 | 10.0 |
| Japan | 9.9 | 3.1 |
| Nigeria | 9.7 | 6.1 |
| Kenya | 9.4 | 9.9 |
| Philippines | 9.2 | 8.5 |
| India | 9.1 | 7.7 |
| Gabon | 7.9 | 3.7 |
| Paraguay | 7.4 | 2.4 |
| Mali | 6.8 | 4.0 |
| Botswana | 6.8 | 5.0 |
| Comoros | 6.7 | 1.9 |
| Ghana | 6.5 | 8.4 |
| Cameroon | 6.1 | 6.1 |
| South Africa | 6.0 | 6.8 |
| Nepal | 6.0 | 1.6 |
| Benin | 5.9 | 9.8 |
| Burkina Faso | 5.1 | 3.7 |
| Liberia | 5.0 | 3.4 |
| Madagascar | 4.8 | 4.2 |
| Mauritania | 4.7 | 8.3 |
| Republic of Congo | 4.7 | 4.1 |
| Thailand | 4.7 | 1.6 |
| Ivory Coast | 4.6 | 4.8 |
| Sierra Leone | 4.5 | 3.1 |
| Uganda | 4.3 | 3.7 |
| Senegal | 3.8 | 2.6 |
| Taiwan | 3.8 | 2.6 |
| Togo | 3.7 | 4.1 |
| Guinea | 3.5 | 5.1 |
| Haiti | 3.3 | 7.6 |
| Rwanda | 3.2 | 2.9 |
| Niger | 3.0 | 8.0 |
| Myanmar | 2.9 | 0.2 |
| Malawi | 2.4 | 6.5 |
| Chad | 2.1 | 5.6 |
| Gambia | 2.1 | 4.1 |
| Afghanistan | 1.9 | 2.5 |
| Indonesia | 1.7 | 4.4 |
| China | 1.6 | 2.5 |
| Laos | 1.4 | 1.4 |
| Pakistan | 1.2 | 4.2 |
| Cambodia | 1.2 | 1.9 |
| Vietnam | 1.0 | 1.3 |

**Fig S7** **Difference in general attitudes to science between high and low science knowledge groups (conditional model)**

| 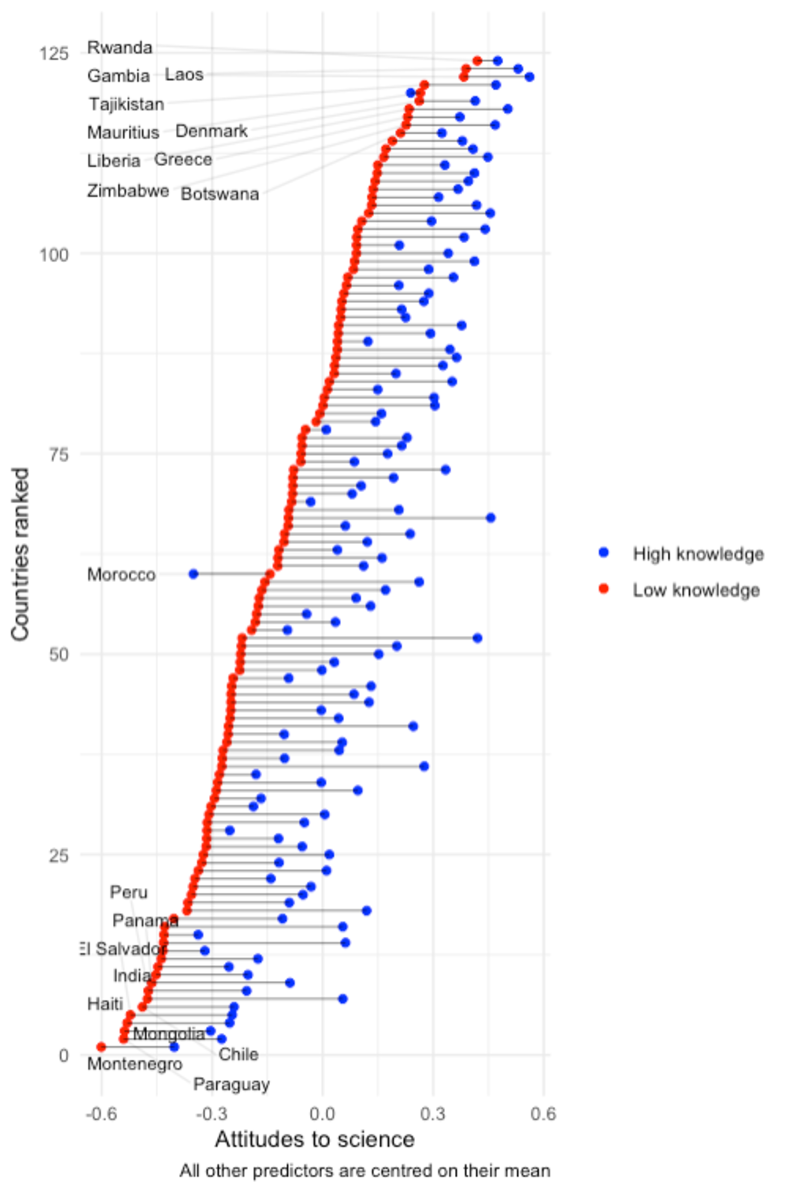 | 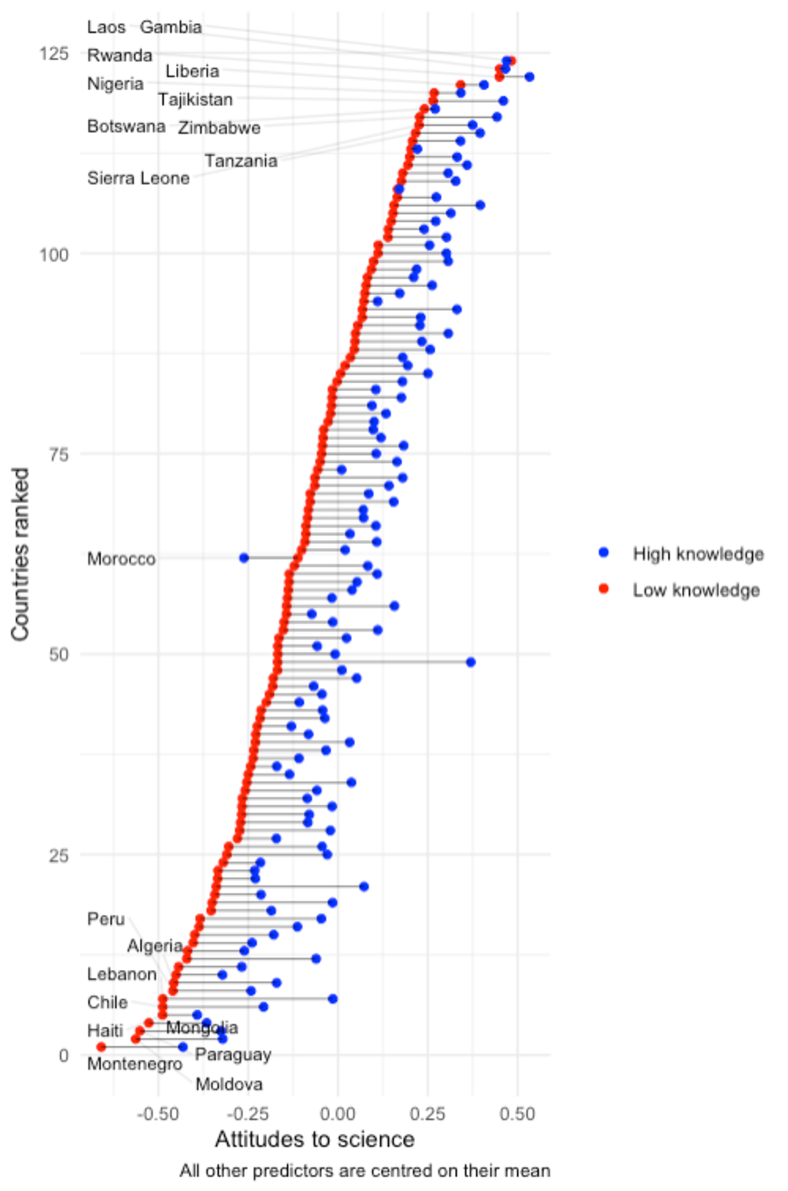 |
| --- | --- |
